# Characteristics of Circulating Fluidized Bed Combustion (CFBC) Ash as Carbon Dioxide Storage Medium and Development of Construction Materials by Recycling Carbonated Ash

**DOI:** 10.3390/ma17174359

**Published:** 2024-09-03

**Authors:** Young Min Wie, Ki Gang Lee, Kang Hoon Lee

**Affiliations:** 1 Department of Materials Engineering, Kyonggi University, 154-42, Gwanggyosan-ro, Yeongtong-gu, Suwon-si 16227, Republic of Korea; supreme98@kyonggi.ac.kr (Y.M.W.); gglee@kyonggi.ac.kr (K.G.L.); 2 Department of Energy and Environmental Engineering, The Catholic University of Korea, 43 Jibong-ro, Bucheon-si 14662, Republic of Korea

**Keywords:** CFBC ash, mineral carbonation process, foamed concrete, waste recycling, carbon dioxide reduction

## Abstract

This study validates the attributes of the mineral carbonation process employing circulating fluidized bed combustion (CFBC) ash, which is generated from thermal power plants, as a medium for carbon storage. Furthermore, an examination was conducted on the properties of construction materials produced through the recycling of carbonated circulating fluidized bed combustion (CFBC) ash. The carbonation characteristics of circulating fluidized bed combustion (CFBC) ash were investigated by analyzing the impact of CO_2_ flow rate and solid content. Experiments were conducted to investigate the use of it as a concrete admixture by replacing cement at varying percentages ranging from 0% to 20% by weight. The stability and setting time were subsequently measured. To produce foam concrete, specimens were fabricated by substituting 0 to 30 wt% of the cement. Characteristics of the unhardened slurry, such as density, flow, and settlement depth, were measured, while characteristics after hardening, including density, compressive strength, and thermal conductivity, were also assessed. The findings of our research study validated that the carbonation rate of CFBC ash in the slurry exhibited distinct characteristics compared to the reaction in the solid–gas system. Manufactured carbonated circulating fluidized bed combustion (CFBC) ash, when used as a recycled concrete mixture, improved the initial strength of cement mortar by 5 to 12% based on the 7-day strength. In addition, it replaced 25 wt% of cement in the production of foam concrete, showing a density of 0.58 g/cm^3^, and the 28-day strength was 2.1 MPa, meeting the density standard of 0.6 grade foam concrete.

## 1. Introduction

Due to concerns about global warming, many researchers are intensively conducting research to reduce CO_2_ emissions [1,2]. There are many areas for reducing CO_2_ emissions, and mineral carbonation is being explored as a promising option for storing CO_2_ [3,4,5]. Mingwei et al. [6] studied mineral carbonation using ophiolite, while Meijssen et al. [7] investigated the carbonation process in concrete. Miao et al. [8] investigated a method of absorbing CO_2_ by carbonating waste incineration ash in an ammonium solution. The basic principle of mineral carbonation is to precipitate the alkali and alkaline earth components present inside the mineral by reacting them with CO_2_. Components such as CaO and MgO are mainly known as raw materials for carbon dioxide (CO_2_) storage. Since ash generated by circulating fluidized bed combustion (CFBC) contains a high amount of free CaO, research on carbon dioxide capture using this ash is also being conducted [9,10,11]. Wee et al. [9] technically reviewed the carbonation process of coal fly ash by dividing it into wet and dry processes, and Kim et al. [10] investigated carbon dioxide storage utilizing CFBC bottom ash. Bae et al. [11] confirmed that the carbonation rate increases linearly with the amount of Ca(OH)_2_ present in CFBC ash.

Coal ash is an industrial by-product generated from coal-fired power plants, and approximately 9 million tons of coal ash is generated annually in Korea. Most of this is coal ash generated through pulverized coal combustion (PCC). However, recently, the construction of CFBC-type boilers has increased, along with their advantage. CFBC boilers are gradually increasing worldwide because they are recognized as a clean thermal power generation with the advantages of excellent combustion efficiency and low environmental load by using a method of combustion by fluidizing solid particles [12,13]. Fly ash and bottom ash are generated in a PCC boiler and, although it is somewhat fluid, the ratio is generally about 80:20 [14,15,16]. In the case of ash generated from PCC boilers, high-temperature stable phases such as silica-alumina generally account for most of it [17,18,19]. Therefore, it is mostly used as a cement admixture. However, CFBC ash generally shows differences in chemical composition compared to PCC ash [20,21]. This is because, in the PCC process, desulfurization is conducted wet by using a separate desulfurization facility but, in the CFBC process, desulfurization is conducted inside the furnace by spraying CaO into the furnace. Therefore, CFBC ash contains a large amount of free CaO and anhydrous gypsum (CaSO_4_). When free CaO and anhydrous gypsum are mixed with water to produce concrete, they induce the formation of Ca(OH)_2_ and ettringite, which can lead to excessive expansion and cracking of concrete [22]. For this reason, unlike PCC ash, its use as a concrete admixture is quite limited.

Various studies, including those in the construction field, have been conducted on the recycling of CFBC ash. Chi [23] described the characteristics of cement composite materials made from recycled CFBC ash and pulverized blast furnace slag and stated that, as the content of CFBC ash increases, the strength decreases and the expansion rate increases. Wu et al. [24] attempted to apply it to autoclave breathable concrete. Jang et al. [25] conducted a study on using CFBC ash as a binder for low-strength materials. In addition, there have been attempts to recycle it into construction materials in various studies [26,27,28,29,30,31]. Additionally, existing studies have shown that it can be recycled using its chemical properties, such as reinputting it into the desulfurization process [32] and using it as a soil stabilizer [33]. In addition, various studies have been conducted recently to recycle fly ash, including porous zeolite synthesis [34], recycling as concrete admixture [35], and alkaline activators [36].

As such, CFBC ash has great recycling potential due to its physical and chemical properties. However, because it has a fundamentally different composition from the PCC process, its use as a construction material is limited. And although various recycling methods have been proposed, they have not yet reached the practical stage. Therefore, CFBC ash is currently rarely recycled and is mostly landfilled or disposed of [37].

In existing research, the carbonation process and recycling into building materials are recognized as separate fields and, therefore, not much research has been conducted on applying carbonated CFBC ash to building materials. However, to recycle CFBC ash into concrete, stabilization of free CaO is essential. The carbonation process stabilizes free CaO, prevents the expansion of concrete, and absorbs CO_2_. For this reason, the product is possible as a building material and the entire process is environmentally friendly.

To solve these problems, this study experimentally optimized the process variables of the wet carbonation process of circulating fluidized bed ash, such as CO_2_ flow, carbonation time, and solid content. Through this, CO_2_ absorption and stabilization of free CaO inside CFBC were achieved. We also investigated the use of CFBC ash carbonated through a wet carbonation process as a concrete admixture and a cement substitute for foamed concrete. Through this process, we aim to contribute to CO_2_ reduction and resource circulation by storing CO_2_ and proposing a method of recycling carbonated CFBC ash as a construction material.

## 2. Materials and Method

### 2.1. Raw Materials

**Figure 1:**
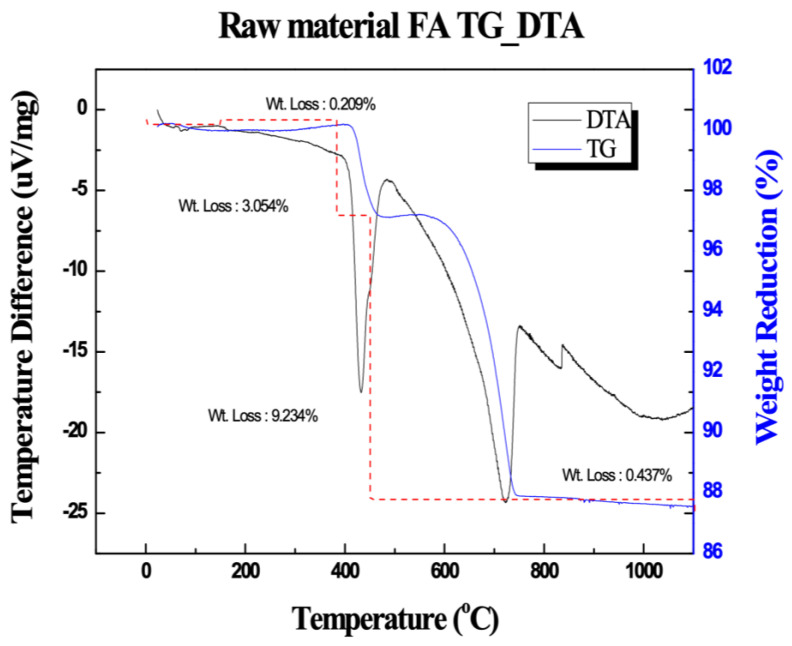
TG−DTA measurement results of CFBC ash.

The chemical composition of CFBC ash, OPC, and slag cement used in this experiment was analyzed through X-ray fluorescence (XRF) (ZSX-100e, Rigaku, Osaka, Japan) (Table 1), which showed that the primary components of CFBC ash were CaO and SiO_2_. The reason why the CaO content appeared high is because, as explained above, the desulfurization process takes place inside the furnace during the CFBC process, so a large amount of limestone is input and it is detected together with the ash. The reason CaO was present at 38.35 wt% and the S component was detected was because of the gypsum produced because of the desulfurization process. The chemical composition of OPC and slag cement falls within the range of commonly commercialized products. The TG-DTA measurement results are shown in Figure 1. As a result of the measurement, there was a weight loss twice, and the weight loss occurring at around 500 °C is due to the decomposition of Ca(OH)_2_. It is known that Ca(OH)_2_ decomposes into CaO and H_2_O at 500 °C [38]. Weight loss at around 720 °C is due to the decomposition of CaCO_3_ [39].

### 2.2. Carbonation of CFBC Fly Ash

**Figure 2:**
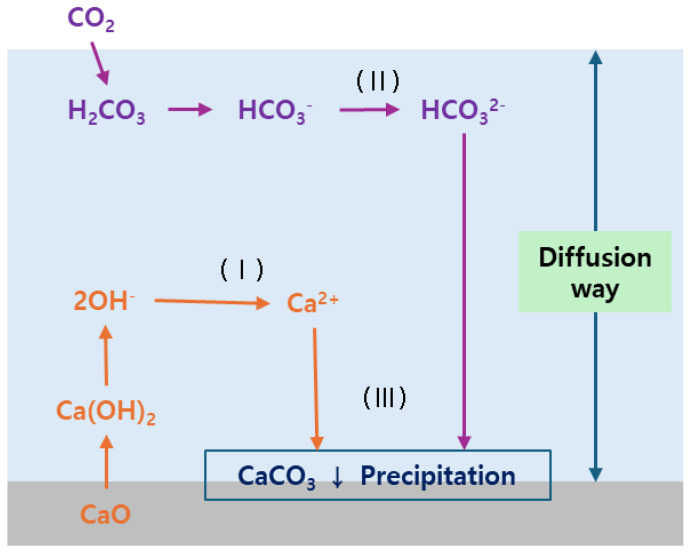
Schematic diagram of carbonation reaction of CaO [11].

We designed an experiment to investigate the carbonation characteristics of CFBC ash and conducted an experiment to produce precipitated calcium carbonate by injecting high-purity 99% CO_2_ gas into a CFBC ash slurry. A carbonation reaction was induced by injecting CO_2_ gas into a slurry mixed with solids and water at a ratio of 1:5 in a plastic beaker with a volume of 2000 mL. The carbonation process of CaO is schematically shown in Figure 2. Bae et al. [11] mentioned the process in which free Cao is carbonated in suspension. The free CaO component is hydrated in the slurry to become Ca(OH)_2_ and dissociates into Ca^2+^ and OH^−^ ions (reaction I). During this process, the pH of the solution increases but the solubility of Ca(OH)_2_ is limited, so the pH ranges from 12 to 13. When CO_2_ gas is injected into this suspension, it is hydrated into H_2_CO_3_ and dissociates into H^+^ ions and CO_3_^2−^ (reaction II). At this time, the H^+^ ions generated react with OH^−^ ions to become H_2_O, and the Ca^2+^ ions react with CO_3_^2−^ ions to produce CaCO_3_ precipitates (reaction III). After this process is completed, the slurry changes from slightly acidic to neutral. At this time, if the solid–liquid ratio is small, the carbonation reaction slows down due to insufficient diffusion distance, so it is necessary to select an appropriate solid–liquid ratio. The solid-to-liquid ratio was selected by referring to Bae et al.’s experiment. To enhance the reaction of solids in the suspension, it was stirred using a magnetic stirrer. Ca(OH)_2_ dissolves in water and is strongly basic but, when it reacts with CO_2_ and precipitates CaCO_3_, it becomes neutral, close to pH 7. This reaction is an exothermic reaction and the temperature rises when it occurs. Therefore, we measured the pH and temperature of the slurry to check if the carbonation reaction was completed. Juverka et al. [40] summarized the carbonation process of CaO in the equation shown in Table 2. According to this, the carbonation process of CaO is an exothermic reaction overall and, because OH^−^ is generated when the hydrate dissociates in water, it has basicity in the intermediate process. Thermodynamically, the sign of ΔG in R (4) to (6) is negative (−), which means that this is a forward reaction due to a spontaneous process, and the activation energy that causes the reaction is low, so the reaction proceeds quickly. However, in the case of R (2) to (3), the sign is positive (+) and the reaction does not proceed quickly because it is challenging to cause the reaction as an involuntary reaction. It is confirmed through thermodynamic properties that R (2) to (3) are rate-limiting steps. The experimental conditions are shown in Table 3. First, to confirm the appropriate CO_2_ flow rate, a carbonation reaction experiment was performed while the flow rate was changed to 100, 300, 500, 700, and 1000 cc/min. As a result of that experiment, another carbonation experiment, one of Ca(OH)_2_ and CFBC ash, was performed by fixing the flow rate at 700 cc/min, which is the optimal flow rate, and the pH and temperature of the slurry were measured.

### 2.3. Recycling Experiment with Concrete Admixture

To confirm the possibility of using carbonated CFBC ash as a concrete admixture, the activity, stability, and setting time were measured. Each experiment was performed according to the ASTM standard, the Korean standard. The activation level was tested by referring to ASTM C618 [41]. The mixing ratio of the mortar used in the experiment is shown in Table 4. The degree of activation is determined according to the compressive strength ratio between the reference sample and the test sample, and the calculation formula is as follows.

(1) $$A=\frac{C_{2}}{C_{1}}\times 100$$

A:activationindex;
$C_{1}:Compressivestrengthofreferencesample$;
$C_{2}:Compressivestrengthoftestsample$.

The density of the admixture was measured following the cement density measurement method specified in ASTM C188 [42] and the stability and setting time of the tested mortar were measured according to KS L ISO 9597 [43]. The stability of cement refers to the degree of volumetric expansion of mortar during curing and, if the stability is low due to the large amount of free CaO, cracks may occur in the concrete. The setting time was measured using a Vicat device, and the initial setting time and ending time were measured.

### 2.4. Manufacturing of Foam Concrete

**Figure 3:**
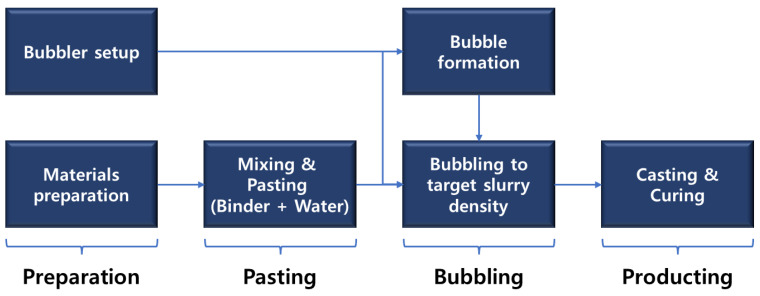
Foamed concrete manufacturing process.

Foam concrete was manufactured to confirm the recycling characteristics of stabilized CFBC ash. The manufacturing process is shown in Figure 3 and the mixing ratio of the foam concrete is shown in Table 5. The mix was designed based on cement and gypsum, replacing cement with CFBC ash up to 30 wt%. The admixture used at this time was a standard AE agent manufactured by SILKROAD C&T CO. LTD. (Seoul, Republic of Korea). The CaSO_4_ used was a product with 99% purity. And the slag cement used was a type 1 product containing slag of 30 wt% or less.

### 2.5. Physical Properties of Cement Pastes

#### 2.5.1. Density of Cement Pastes

The physical properties of the cement paste were measured in terms of slurry density, settlement depth, and actual void ratio. The slurry density was tested according to the manner specified in ASTM D4380 [44], which is as follows. The collected slurry was poured into a separately manufactured 1000 mL container to the top. Then, the remaining upper part was removed horizontally and the mass (WS) of the sample excluding the mass of the container was measured to the nearest 1 g. The density of the slurry is calculated using Equation (2).

(2) $$Densityoffoamslurry=\frac{W_{s}}{1000}$$

$W_{s}$: mass of sample(g);
1000 mL: volume of container.

#### 2.5.2. Flow and Sinking Depth of Cement Paste

The method of measuring slurry flow was as follows. An acrylic cylinder with an inner diameter of 80 mm and a height of 80 mm was placed on a 350 mm × 350 mm glass plate. After that, the slurry sample was poured into the top, the remaining top was removed horizontally, the cylinder was lifted, and, after 1 min, the average value of the lengths measured in the four directions in which the sample spread was the flow value. The sinking depth was obtained by pouring the sample to the top of a transparent acrylic container with an inner diameter of 145 mm and a height of 300 mm, removing the remaining upper part horizontally, and measuring the top sinking depth 2 h later.

### 2.6. Measurement of Absolute Dry Bulk Density and Compressive Strength of Foamed Concrete

The absolute dry bulk density and compressive strength of cured concrete were tested using the test method of KS F 2459 [45]. The mixed concrete specified in Table 5 was molded into a cylinder with a diameter of 100 mm and a height of 200 mm. After curing at a temperature of 20 ± 2 °C for 48 h, the upper part of the specimen was flattened and the mold was demolded. Then, it was cured in water for 28 days. The cured specimen was dried at a constant weight at 105 ± 5 °C, cooled to room temperature, and then weighed (W_0_). Then, the volume (V) was measured and the apparent density was calculated as follows, and the compressive strength of the completed samples was measured.

(3) $$Absolutedrybulkdensity=\frac{W_{0}}{V}$$

$W_{0}:Weightofdriedspecimen$;
V:Volumeofdriedspecimen.

### 2.7. Measurement of Thermal Conductivity of Foamed Concrete

A specimen with a size of 300 mm × 300 mm × 50 mm was manufactured and the thermal conductivity was measured. In the test, the thermal conductivity was calculated by measuring the surface temperature at an average temperature of 21 ± 3 °C and with the heat flow direction facing upward, based on KS L 9016 [46]. The equation for calculating thermal conductivity is as follows:

(4) $$Thermalconductivity=\frac{l}{R_{C}}(W/m\cdot k)$$

(5) $$R_{C}=\frac{A\cdot (\theta_{1}-\theta_{2})}{P}$$

l:Thicknessoftestspecimen(m);
$R_{C}:Thermalresistanceofthetestspecimen(m^{2}\cdot K/W)$;
$A:Effectiveareaofmainheatingplate(m^{2})$;
$\theta_{1}:Temperatureofhotsideoftestspecimen(K)$;
$\theta_{2}:Temperatureofcoldsideoftestspecimen(K)$;
P:Powersuppliedtothemainheatingplate(W).

## 3. Results and Discussion

### 3.1. Carbonation of CFBC Ash

**Figure 4:**
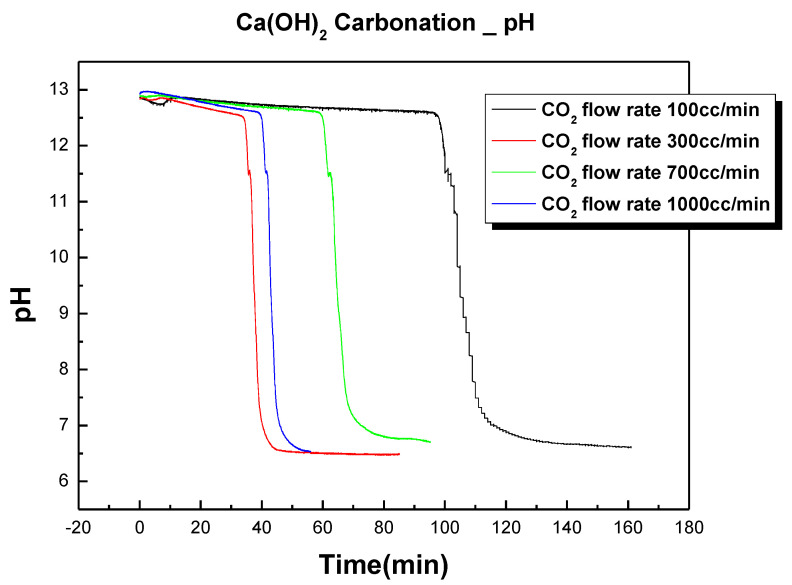
pH change of slurry due to CO_2_ flow rate change.

**Figure 5:**
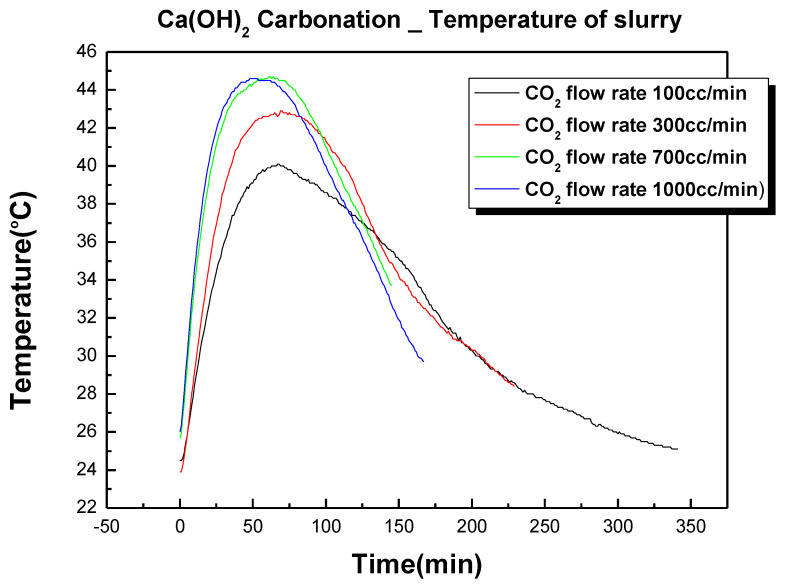
Temperature change of the slurry due to CO_2_ flow rate change.

In experiment (1) of Table 3, Ca(OH)_2_ was slurred at a solid–liquid ratio of 1:5 and a carbonation experiment was conducted by changing the CO_2_ flow rate in four ways from 100 to 1000 cc/min. Figure 4 is the experimental result showing the change in pH according to the change in the CO_2_ flow rate. Figure 5 shows the temperature change due to the exothermic reaction that occurred during this process. The carbonation experiment confirmed that, as the CO_2_ flow rate increased, the carbonation reaction completion time decreased. This was because, as the CO_2_ flow rate increased, the amount of CO_2_ dissolved in the slurry increased, which promoted the reactions in Equations (3) and (4) in Table 2. As the CO_2_ flow rate increased, the carbonation reaction completion time decreased but it no longer increased significantly above 700 cc/min because the saturated dissolved state of CO_2_ was reached under the conditions of this experiment at 700 cc/min. The temperature rise due to the exothermic reaction was almost identical under flow rate conditions of 700 cc/min and 1000 cc/min, which suggested that the reaction speed no longer increased under flow conditions above 700 cc/min.

**Figure 6:**
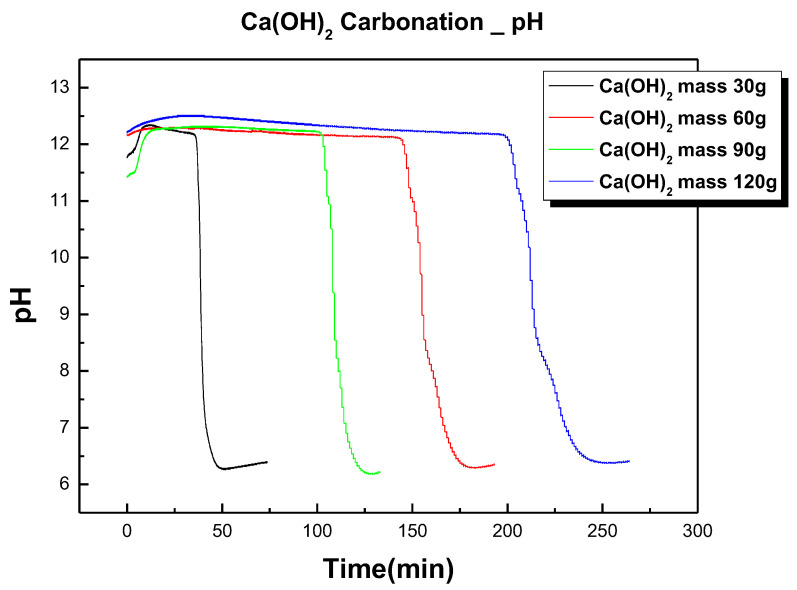
pH change with Ca(OH)_2_ mass change in slurry.

Through the previous experiment, we were able to confirm the CO_2_ flow conditions in the carbonation process. To confirm the carbonation reaction rate according to the change in the amount of solids in the slurry, an experiment designed in number (2) of Table 3 was conducted. Figure 6 shows the pH changes of the prepared Ca(OH)_2_ slurry over time. As Ca(OH)_2_ increased, the reaction completion time increased linearly. It is widely known that the gas and solid phase reaction between CaO and CO_2_ proceeds in two rate-limiting regions. At the initial stage of the reaction, the reaction progresses at a very fast rate due to a nonuniform surface reaction but, as time passes and CaCO_3_ is densely formed on the surface, the diffusion rate of CO_2_ slows down significantly and the reaction rate decreases. Kingerry et al. [47] presented the reaction rate equation for when the reaction proceeds in cylindrical particles as follows:

(6) $$(1-\alpha {)}^{\frac{1}{2}}=1-kt/r_{0}$$

α:Decompositionrate;
k:Reactionrateconstant;
t:Reactiontime;
$r_{0}:Initialparticleradius$.

**Figure 7:**
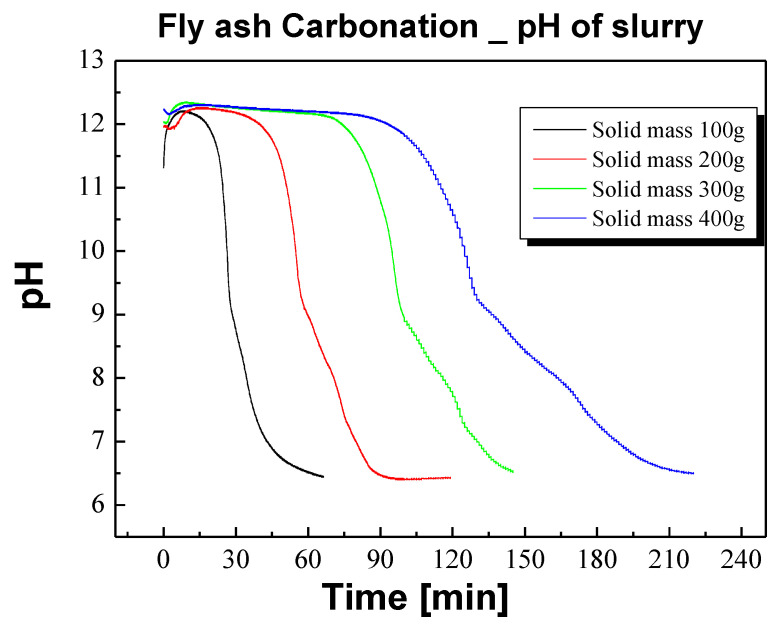
pH changes of slurry according to fly ash solid content change.

Our experimental results were different from the previous equation because the reaction in the experiment was not a reaction between a gas and a solid but a reaction in a slurry. Hwang et al. [48] described the carbonation reaction of lime milk and, in this wet aqueous solution reaction, the Ca dissolution reaction and CO_2_ absorption reaction corresponding to reactions (2) and (3) in Table 2 were rate-dependent reactions. Therefore, even if the rate of the Ca dissolution reaction was increased by increasing the amount of Ca in the experimental design, the reaction rate remained constant because the flow rate of CO_2_ was constant. Therefore, when the amount of solid increases, the reaction completion time increases linearly. We confirmed that this trend also appears in the carbonation process of actual CFBC ash (Figure 7). 

### 3.2. Pilot Test for Carbonation of CFBC Ash

**Figure 8:**
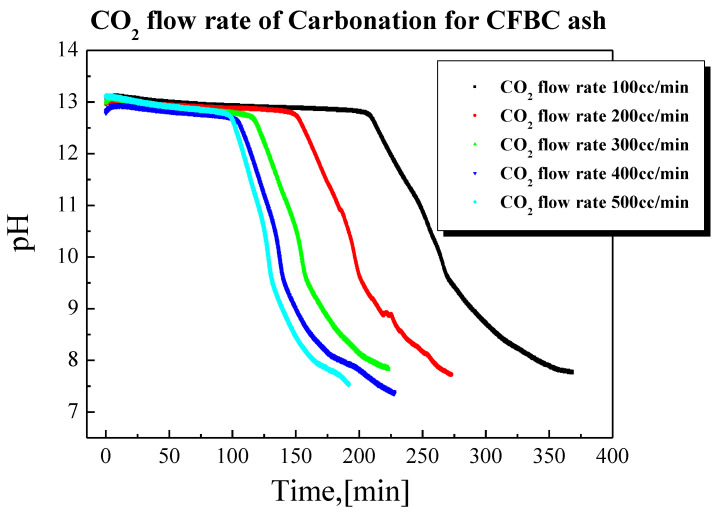
pH change with carbonation time.

The mineral carbonation process requires a subsequent drying process because a large amount of water is input. Therefore, for commercialization, it is very important to reduce the amount of water used. As a preliminary experiment to reduce the amount of water used in the carbonation process and scale up to the pilot size, a carbonation experiment was conducted using 300 g of CFBC ash at a solid-to-liquid ratio of 1:2. The performed experiments are shown in Table 6 and the experimental results are shown in Figure 8. The results of the experiment confirmed the saturated dissolution flow rate at a CO_2_ flow of 300 cc/min.

**Figure 9:**
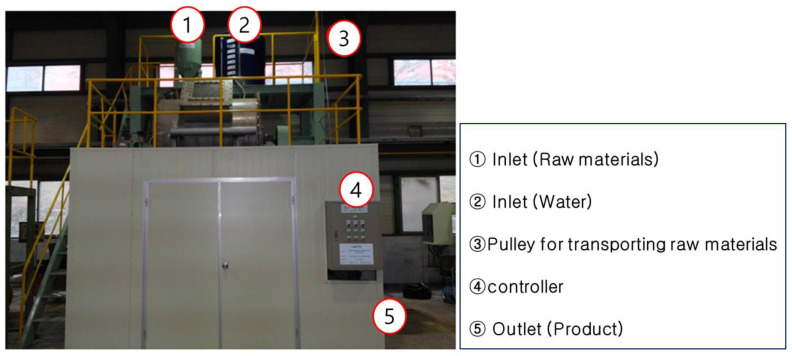
Pilot equipment for carbonation of CFBC ash.

**Figure 10:**
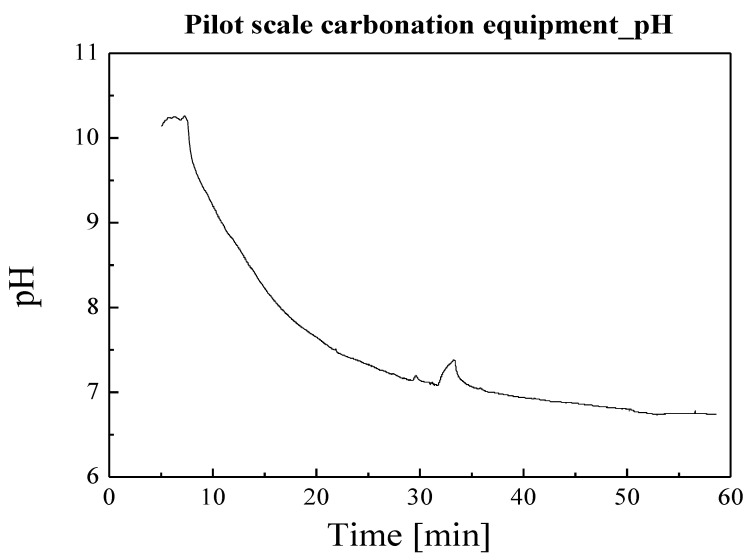
pH changes in pilot scale carbonation process.

**Figure 11:**
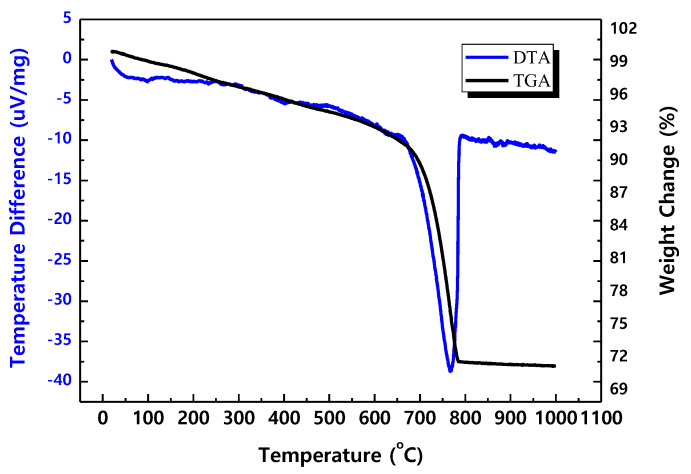
TG−DTA analysis of fly ash after carbonation reaction.

In order to scale up the carbonation process of CFBC ash, the carbonation process was performed in a pilot scale facility. The pilot equipment used in the experiment is shown in Figure 8. During the experiment, the solid-to-liquid ratio was set to 1:2. The experiment was conducted with the pressure of CO_2_ introduced for carbonation at 0.05 MPa and the amount of CFBC ash was 30 kg. The pH change according to reaction time is shown in Figure 9, which confirmed that the pH was maintained at around 10 to 10.5 for the first 5 min and then slowly decreased. After the carbonation process was conducted for 1 h, the pilot test was completed, and the TG-DTA measurement results of the carbonated CFBC ash are shown in Figure 10. Before the carbonation process, weight loss at around 500 °C was confirmed but it was not detected in CFBC ash after the carbonation process was completed at pH 7. A very large weight loss occurs around 800 °C, which we think is a decomposition phenomenon of CaCO_3_. Wei et al. [49] experimentally showed that, when CaCO_3_ was heated in an electric furnace, weight loss occurred intensively at 750–800 °C, similar to Figure 11. 

### 3.3. Evaluation of the Potential of CFBC Ash as a Concrete Admixture

Various inorganic admixtures are used to reduce the amount of cement in concrete and improve its physical properties [50]. Admixtures such as PCC fly ash and silica fume are mixed in various ways to increase the strength of concrete [51,52]. PCC fly ash is known to be used to increase the durability and formability of concrete and reduce permeability [53]. Moreover, Chishi et al. [54] stated that silica fume increases the mechanical strength of concrete. The strength of cement is strengthened through the pozzolanic reaction, and the role of Ca(OH)_2_ is essential in the pozzolanic reaction. Recently, CaCO_3_ cement technology for carbon dioxide capture has been studied [55,56]. According to Matschei et al. [56], CaCO_3_ can be used as a concrete mixture, either as an inert filler or as a reactive mixture. Since carbonated CFBC ash contains a large amount of CaCO_3_, it is thought to be able to contribute to the improvement of concrete strength to some extent. CaCO_3_ partially dissolves in water to become Ca(OH)_2_, which promotes the pozzolanic reaction. This means that the addition of CaCO_3_ can promote the pozzolanic reaction of the structure. A study by Cosentino et al. [57] reported that CaCO_3_ nanopowder increased the initial strength of mortar. 

We manufactured mortar to check the potential of carbonated CFBC ash as a concrete admixture. The activity index of mortar is shown in Table 7. Samples in which OPC was substituted showed a somewhat lower activity index at 14 days. On the other hand, experiments in which slag cement was replaced confirmed that it had a good activity index exceeding 100. Although the activity index of mortar substituted with carbonated admixture is somewhat insufficient to replace OPC, it is expected to improve the initial strength of concrete using slag cement because it has a higher activity index than slag cement. The density, stability, setting time, etc., of the mortar are shown in Table 8. We confirmed that the density decreased somewhat as the amount of substitution increased. However, the stability was found to be at the same level as that of regular concrete and the setting time was also similar. The experimental results show that the values of initial and final setting times are approximately 40 to 60 min but the difference is generally in the range of 120 to 240 min. However, this value is very sensitive to curing conditions and is influenced by the judgment of the experimenter. Therefore, it is thought that the somewhat smaller values are due to this experimental error. We think that up to 20 wt% can be substituted in the blast furnace slag cement system without deteriorating physical properties. The strength of concrete is closely related to the pozzolanic reaction. It is well known through previous research that CaCO_3_ promotes the pozzolanic reaction. The pozzolanic reaction is a very complex reaction of CaO–SiO_2_–H_2_O. Therefore, the reactivity does not increase linearly as the amount of CaCO_3_ increases. Therefore, it is judged that the content of CFBC ash is limited.

### 3.4. Foamed Concrete

#### 3.4.1. Physical Properties of Foamed Concrete Paste

**Figure 12:**
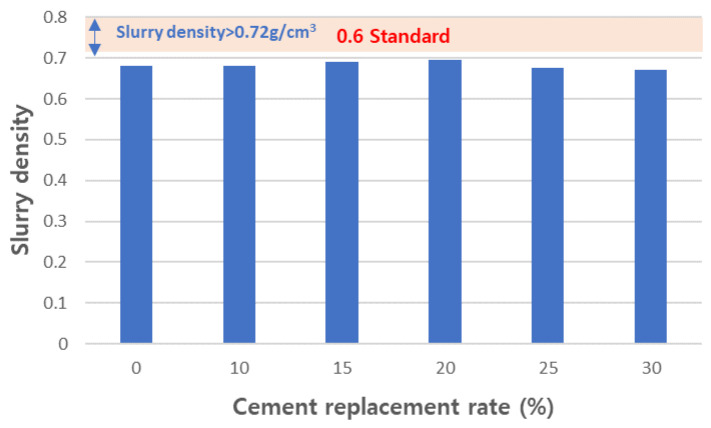
Density of test slurries.

**Figure 13:**
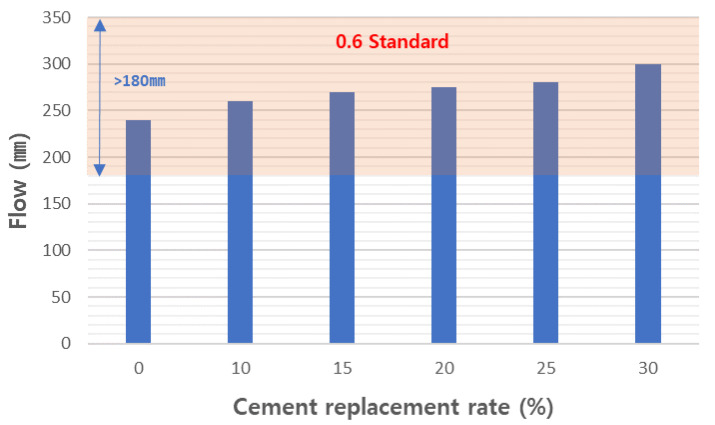
Flow of test slurries.

**Figure 14:**
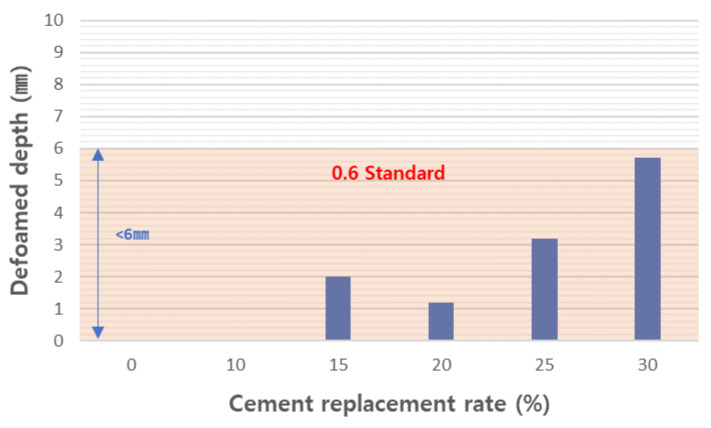
Settlement depth of test slurries.

To determine the working properties of foamed concrete, we measured the density and flow of the tested mortar samples. The slurry densities of the experimental mixtures were measured and are shown in Figure 12. Slurry density is treated as an important characteristic for designing the density of foamed concrete products. This means that, to produce foam concrete that meets the specifications, it is important to have a slurry density that meets the specifications. KS F 4039 [40] defines the quality standards for cement paste for pouring foamed concrete on site, and the 0.6 grade foamed concrete standard has a slurry density of 0.72 g/cm^3^ or more. The slurry densities of the tested mixtures were manufactured to be slightly lower than that at a range of 0.67 to 0.695. The slurry flow was measured and is shown in Figure 13. In foamed concrete, slurry flow is an important characteristic that greatly affects not only the molding workability of the slurry but also the physical properties of the final concrete. This slurry flow must be designed so that bubbles are evenly distributed throughout the product. If this flow is too low, workability will deteriorate and bubbles will become uneven, which will hinder the reliability of the final product. Therefore, KS F 4039 [40] specifies the slurry flow as 180 or more. All formulations designed in this experiment met this standard. As the amount of CFBC ash substitution increased, the slurry flow tended to increase. Figure 14 shows the measured sinking depth of the slurry. It is difficult to completely control the sinking phenomenon of slurry when manufacturing foam concrete. However, if the sinking depth is deep, it may have a negative effect on the physical properties of the concrete after curing, such as the strength of the concrete and the homogeneity of pore distribution, so it needs to be managed to remain below a certain level. In KS F 4039 [40], the standards are different for each grade but, for 0.6 grade foamed concrete, the standard is 6 mm or less. We found that the higher the substitution rate, the greater the sinking depth and, in the case of the 30 wt% substituted product, it was almost 6 mm. However, all the samples met the standards and we believe that the standards will be exceeded when CFBC ash is replaced by more than 30 wt%. Therefore, we consider it appropriate to limit the substitution amount of CFBC ash to 30 wt% or less. In general, the settlement phenomenon of aerated concrete occurs due to the defoaming phenomenon, and this is affected by the cohesion between air bubbles and the decrease in the viscosity of the cement slurry [58]. It is known that the increase in the viscosity of cement slurry is more advantageous for raw materials with high fineness [59]. In this way, the defoamed depth of the slurry is very closely related to the viscosity of the slurry. In general, the viscosity of the slurry can be indirectly inferred through the flow, and the flow increases as the amount of CFBC ash added increases. This means that the viscosity decreases as the amount of addition increases. Therefore, it can be seen that the settlement depth increases as the amount of CFBC ash added increases. There are various reasons for this decrease in viscosity but it is said that it is greatly affected by the fineness of the physical properties of the raw materials [60]. Therefore, it is presumed that the settlement depth increases as the amount of addition increases because the fineness of the raw material, CFBC ash, is low and, because of this, the settlement depth increases as the substitution amount increases and it is thought that the substitution amount is limited due to this phenomenon.

#### 3.4.2. Physical Properties of Cured Concrete

**Figure 15:**
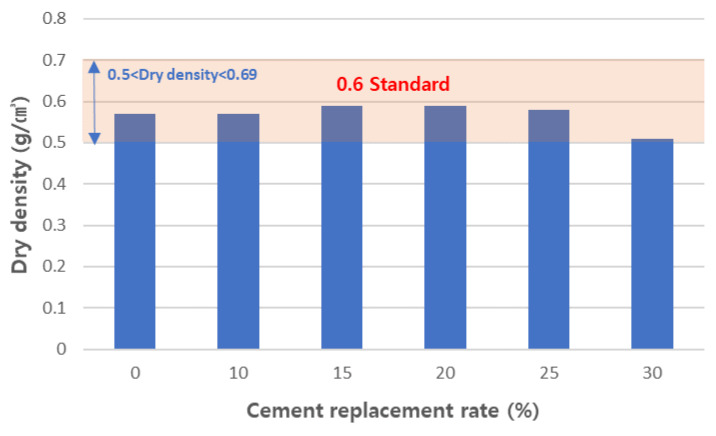
Dry density of aerated concretes.

**Figure 16:**
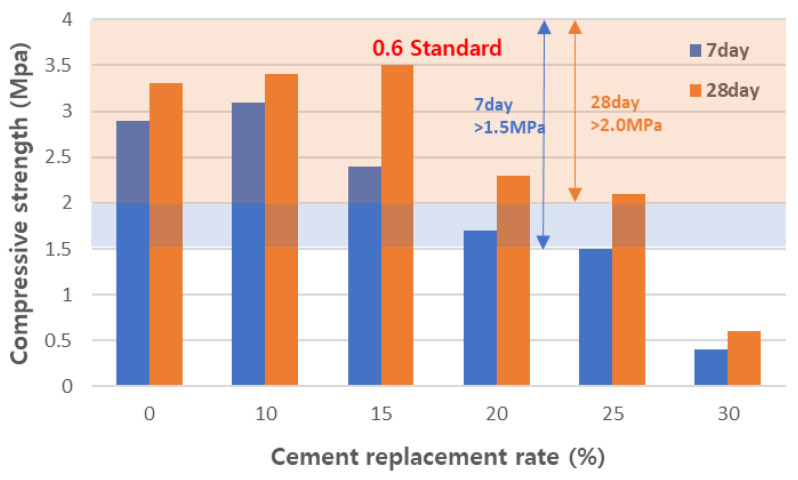
Compressive strength of aerated concretes.

**Figure 17:**
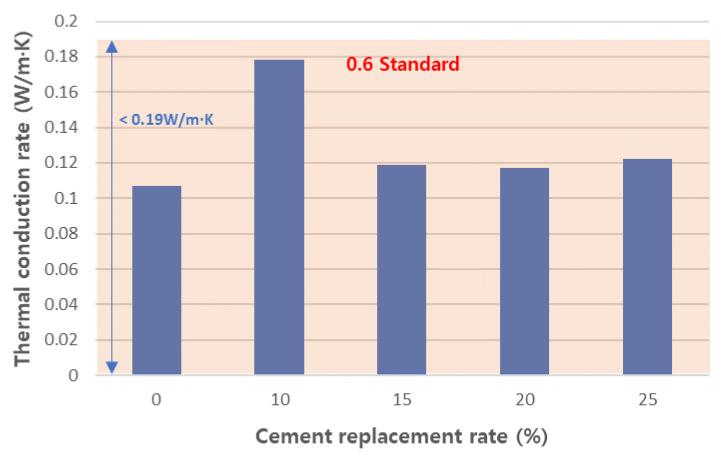
Thermal conduction rate of aerated concretes.

We measured the apparent density of the foamed concrete mix samples and the results are shown in Figure 15. We confirmed the apparent density of the cured foamed concrete to meet the target standard of 0.6-grade foamed concrete. The density standard for 0.6-grade foamed concrete was 0.5–0.7 g/cm^3^, which all the mixes sufficiently satisfied. Since foamed concrete is mainly used as a nonstructural insulation material, it does not require high strength. However, an appropriate level of strength is required to maintain shape and quality. The 7-day and 28-day compressive strengths of the samples were measured and are shown in Figure 16. When the amount of CFBC ash substitution was more than 15 wt%, the compressive strength tended to decrease. However, we found that up to 20 wt% of CFBC-ash-substituted samples satisfied the KS F 4039 standard. As a result of compressive strength measurement, we believe that it is possible to stably replace CFBC ash up to 15 wt%, but the low initial strength should be taken into account when curing. Since foamed concrete is mainly used as an insulating material, its heat conduction properties are very important. In KS F 4039, the standard for thermal conductivity of 0.6-grade foamed concrete is 0.19 W/m·K or less. Figure 17 shows the measured thermal conductivity of the designed formulations. All the samples met the criteria. The density and thermal conduction rate of the material are inversely proportional. However, the experimental results did not show a significant correlation. In this case, it may be due to differences in materials and the curing process. In general, it was said that the nonuniformity of the foam structure of foam concrete increases thermal conductivity and, when the thermal conductivity of the basic material is high, the thermal conductivity can increase. Also, when the moisture content is high or proper curing is not performed during the curing process, the thermal conductivity increases. In this case, when the substitution amount was 30%, the dry density was relatively lower than other specimens, but there was no significant difference in thermal conductivity. This suggests that, when the substitution amount increases, the homogeneity of the raw material decreases and the foam structure becomes irregular. Therefore, increasing the substitution amount by more than 25% significantly reduces the strength and has no advantage in terms of thermal conductivity, so it is reasonable to keep the substitution amount below that. 

## 4. Conclusions

This study confirmed the mineral carbonation conditions of CFBC ash, and the carbonated CFBC ash was used as a concrete admixture and foamed concrete for recycling, and the product properties were confirmed and the following conclusions were obtained:

1. In the carbonation experiment of CFBC ash, the saturation concentration was reached under the condition of 700 cc/min and the optimal carbonation speed was obtained and the reaction rate did not increase, even at a higher CO_2_ flow.
2. As the amount of CFBC ash increased, the carbonation time increased linearly. This suggests that the dominant reaction of the mineral carbonation reaction in the slurry was a dissolution of each ion and has characteristics different from the solid–gas system.
3. When recycling carbonated CFBC ash as a concrete admixture, we confirmed that up to 20 wt% could be replaced in the blast furnace slag cement system and that the initial strength in the blast furnace slag cement system could be improved.
4. As the content of CFBC ash increases, the flow increases, which means that the viscosity decreases. The decrease in viscosity increases the sinking height and worsens the pore structure, so the replacement amount should be limited to 30 wt% or less.
5. As the amount of added carbonated CFBC ash increased, the sinking depth increased when foamed concrete was manufactured, so the replacement amount was limited to 30 wt% or less.
6. Since a rapid decrease in density and strength occurs when the substitution amount is 30%, the replacement amount should be limited to 25 wt% or less.
7. When carbonated CFBC ash was recycled into foamed concrete, it was possible to manufacture foam concrete that satisfied KS F 4039 by substituting up to 25 wt% of OPC.

This study confirmed some process conditions for the carbonation of CFBC ash and confirmed the possibility of recycling carbonated CFBC ash into concrete. Carbonated CFBC ash is expected to be usable as a concrete admixture through the stabilization of free CaO, and foamed concrete using recycled CFBC ash was able to achieve results that meet Korean standards. The results of this experiment can theoretically be applied to the recycling of wastes containing a large amount of free CaO in addition to CFBC ash, and this study can be used to study the recycling of other wastes with similar characteristics.

## Figures and Tables

**Table 1:** Chemical composition of CFBC ash (%).

	ig-Loss	SiO_2_	Al_2_O_3_	Fe_2_O_3_	CaO	MgO	Na_2_O	K_2_O	TiO_2_	P_2_O_5_	MnO	C	S	Total
CFBC ash	1.05	24.73	10.33	4.95	38.35	4.54	4.03	0.84	0.51	0.13	0.00	0.32	10.15	99.94
OPC	2.45	20.03	5.40	2.87	64.35	3.42	0.35	0.54	0.30	0.11	0.11		-	99.93
Slag cement	0.9	29.42	11.12	1.27	51.55	4.27	0.24	0.27	0.66	0.02	0.21	-	-	99.93

**Table 2:** Reaction and chemical potential energy of carbonation [40].

Carbonation Chemical Reaction in Water	ΔG (Unit: KJmole^−1^)		Reaction
CaO (s) + H_2_O (l) → Ca(OH)_2_ (s) Ca(OH)_2_ (s) + H_2_O (l) → Ca^2+^ (aq) + 2OH^−^ (aq) + H_2_O (l)	−57.83 30.42	R. (1) R. (2)	(I) Ca ionization
CO_2_ (g) + H_2_O (l) → CO_2_ (aq) + H_2_O (l) CO_2_ (aq) + OH^−^ (aq) → HCO_3_^−^ (aq) HCO_3_^−^ (aq) + OH^−^ (aq) → H_2_O (l) + CO_3_^2−^ (aq)	8.38 −43.55 −20.92	R. (3) R. (4) R. (5)	(II) CO_2_ dissolution and ionization
Ca^2+^ (aq) + CO_3_^2−^ (aq) → CaCO_3_ (s)	−47.40	R. (6)	(III) CaCO_3_ Precipitation

**Table 3:** Carbonation experimental conditions.

**(1) Experimental Conditions for Carbonation Reaction According to CO_2_ Gas Flow Rate Change**		
**Ca(oh)_2_ mass**	**Flow Rate of CO_2_ Gas**	**Solid/Liquid Ratio**
50 g	100 cc/min	1:5
	300 cc/min	
	500 cc/min	
	700 cc/min	
	1000 cc/min	
**(2) Conditions for the Carbonation Reaction Experiment According to the Solids Amount Changes**		
**Solid (CFBC ash/Ca(OH)_2_)**	**Flow Rate of CO_2_ Gas**	**Solid/Liquid Ratio**
100 g/30 g	700 cc/min	1:5
200 g/60 g		
300 g/90 g		
400 g/120 g		

**Table 4:** Mortar mixing ratio.

Samples	Cement	Ash	Standard Sand	Water
Plain (OPC)	450 ± 2 g	0 g	1350 ± 5 g	225 ± 1 g
Plain (Slag Cement)	450 ± 2 g	0 g	1350 ± 5 g	225 ± 1 g
R10	405 ± 2 g	45 ± 0.5 g	1350 ± 5 g	225 ± 1 g
R20	360 ± 2 g	90 ± 0.5 g	1350 ± 5 g	225 ± 1 g

**Table 5:** Mixing proportions for aerated concrete.

No.	W/B (%)	Unit Binder (kg/m^3^)	Foam Rate (%)	Binder Ratio (%)			Admixture (%)
				Cement	Gypsum	Carbonated CFBC Ash	
1	25	500	65.8	97	3	0	0.4
2			65.0	87		10	
3			64.5	82		15	
4			64.1	77		20	
5			63.7	72		25	
6			63.2	67		30	

**Table 6:** Experimental design of carbonation laboratory test to establish pilot test conditions.

CFBC Ash Mass (g)	Flow Rate of CO_2_ Gas (cc/min)	Solid: Liquid Ratio
300	100	1:2
	200	
	300	
	400	
	500	

**Table 7:** Mortar mixing ratio to foamed concrete.

No.	Sample Name	Cement Replacement Amount	Concrete Activation		
			3 Day	7 Day	14 Day
1	Plain (OPC)	0	100	100	100
2	R10	10	91	85	88
3	R20	20	100	88	89
4	Plain (slag cement)	0	100	100	100
5	R10	10	106	112	99
6	R20	20	106	105	94

**Table 8:** Mortar property measurement experiment results.

No.	Experiment Item	Unit	Plain (OPC)	Plain (Slag Cement)	R10	R20
1	Density	g/cm^3^	3.07	3.03	3.04	2.91
2	Stability	mm	0.5	0	0.5	0
3	Setting time (beginning)	Min	222	216	218	230
4	Setting time (closing)	Min	260	288	275	280

## Data Availability

The original contributions presented in the study are included in the article, further inquiries can be directed to the corresponding author.

## Nomenclature

CFBC	Circulating Fluidized Bed Combustion
PCC	Pulverized Coal Combustion
Anhydrous gypsum	CaSO_4_
XRF	X-ray fluorescence
OPC	Ordinary Portland Cement
